# Foraging on Individual Leaves by an Intracellular Feeding Insect Is Not Associated with Leaf Biomechanical Properties or Leaf Orientation

**DOI:** 10.1371/journal.pone.0080911

**Published:** 2013-11-15

**Authors:** Justin Fiene, Lauren Kalns, Christian Nansen, Julio Bernal, Marvin Harris, Gregory A. Sword

**Affiliations:** 1 Department of Entomology, Texas A&M University, College Station, Texas, United States of America; 2 Biology Department, Elmhurst College, Elmhurst, Illinois, United States of America; 3 School of Animal Biology, the University of Western Australia, Crawley, Western Australia, Australia; University of Arizona, United States of America

## Abstract

Nearly all herbivorous arthropods make foraging-decisions on individual leaves, yet systematic investigations of the adaptive significance and ecological factors structuring these decisions are rare with most attention given to chewing herbivores. This study investigated why an intracellular feeding herbivore, Western flower thrips (WFT) *Frankliniella occidentalis* Pergande, generally avoids feeding on the adaxial leaf surface of cotton cotyledons. WFT showed a significant aversion to adaxial-feeding even when excised-cotyledons were turned up-side (abaxial-side ‘up’), suggesting that negative-phototaxis was not a primary cause of thrips foraging patterns. No-choice bioassays in which individual WFT females were confined to either the abaxial or adaxial leaf surface showed that 35% fewer offspring were produced when only adaxial feeding was allowed, which coincided with 32% less plant feeding on that surface. To test the hypothesis that leaf biomechanical properties inhibited thrips feeding on the adaxial surface, we used a penetrometer to measure two variables related to the ‘toughness’ of each leaf surface. Neither variable negatively co-varied with feeding. Thus, while avoiding the upper leaf surface was an adaptive foraging strategy, the proximate cause remains to be elucidated, but is likely due, in part, to certain leaf properties that inhibit feeding.

## Introduction

For most arthropod herbivores foraging on individual plants requires three hierarchical decisions: which branch to settle on, which leaf to settle on, and where to feed within an individual leaf. For herbivores with fairly immobile immature stages, the mother will determine on which branch and possibly even on which leaf the progeny will feed. However, the final decisions- where to feed on individual leaves- is one that nearly all herbivores ultimately encounter, and yet surprisingly little is known about the factors affecting these foraging-decisions with most attention given to chewing (i.e., mandibulate) herbivores [1-3]. For instance, some chewers avoid foraging on major veins in *Medicago truncatula* (Gaertner) due to higher levels of calcium oxalate crystal [2], and on the periphery and midvein of *Arabidopsis thaliana* (L.) Heynh. leaves due to higher concentrations of allelochemicals such as glucosinolates[3]. Another chewer, *Galerucella lineola* (F.) prefers feeding in the rolled-leaf margins of their host plant, *Salix viminalis* L., due to increased protection from desiccation[1]. Taken together, the environmental conditions associated with particular areas on individual leaves as well as the chemical properties of the leaf itself can influence where chewing herbivores feed on individual leaves. However, comparatively less is known about the factors influencing the within-leaf foraging decisions of intracellular feeding herbivores that consume the cellular contents of host plants. 

One foraging pattern that deserves additional attention is that some intracellular feeding thrips tend to feed from the abaxial leaf surface [4,5]. Two hypotheses to explain this feeding preference have been previously proposed. Fennah (1963) [5] manipulated the orientation of individual leaves in field and laboratory and concluded that the preference for the abaxial leaf surfaces by *Selenothrips rubrocinctus* (Giard) was primarily related to avoidance of direct light (i.e., negative phototaxis) and only secondarily by ‘attractiveness’ of the abaxial leaf surface. Conversely, Wardle & Simpson (1927) [4] examined cross-sections of cotton (*Gossypium hirsutum* L.) leaves noting the upper epidermis was 55% thicker than the lower epidermis and hypothesized that the preference for abaxial feeding by *Thrips tabaci* was unlikely related to negative phototaxis, but rather due to the thickness of the epidermis. 

Thrips mouthparts are characterized as piercing-sucking, and in the sub-order Terebrantia, a typical feeding event involves two steps: 1) the mandible which has a pointed tip but no opening is used to punch a hole into the leaf surface, and 2) the maxillary stylets, which interlock and open at the tip to form the feeding tube, enter the hole created by the mandible and begin to puncture cells and ingest intracellular contents [6]. The mandible is fused proximally to the exoskeleton and cannot be protracted by direct muscular action, resulting in an indirect force generated from moving the whole head downwards and backwards [6]. Therefore, the thickness of the upper epidermis could potentially affect intracellular feeding by increasing the force needed to penetrate with their mandibles through the leaf surface. Similarly, the cellular organization of the palisade layer, which is located ventrally relative to the upper epidermis, could require more force for thrips to penetrate with their mandibles. The palisade layer is comprised of columnar-shaped cells that are more densely packed compared to the spongy mesophyll, which contains irregular-shaped cells surrounded by large air spaces that promote gas-exchange [7]. Thus, intracellular feeding thrips might avoid feeding on the adaxial surface due to the biomechanical properties of that surface which inhibit the quantity of resources consumed and negatively affects relative fitness. 

Because intracellular feeding thrips can potentially consume each plant layer independently (upper epidermis, palisade, spongy mesophyll and lower epidermis), the chemical variation across the dorsi-ventral axis of individual leaves might also affect thrips foraging. For instance, the palisade layer generally has more chloroplast-containing cells and proteins that function in photosynthesis compared to the spongy mesophyll [7]. Such proteins have been considered the primary source of protein in leaves for leaf-mining herbivores [8]. Given thrips seem to avoid feeding from the adaxial surface, we hypothesized that variation in cellular contents may have unique effects on different feeding guilds. 

In this study we investigated why intracellular feeding insects, such as the Western flower thrip (WFT) *Frankliniella occidentalis* Pergande, preferred to feed on the abaxial surface of cotton cotyledons. A preference for abaxial feeding by various species of thrips has been noted previously [4,9,10], but systematic investigations into the causes and consequences of this foraging preference have been limited [5]. Therefore, our goals were to: 1) investigate whether negative phototaxis influences WFT foraging, 2) assess under no-choice conditions the fitness consequences of feeding on each leaf surface, 3) to determine whether leaf surface effects on fitness were due to reductions in the quantity of resources consumed or to post-ingestive effects [11], and 4) investigate the role of leaf ‘toughness’ [12] as a mechanism that inhibits thrips feeding on the adaxial surface. 

## Results

### WFT feeding preferences (choice) and effects of leaf orientation

**Figure 1:**
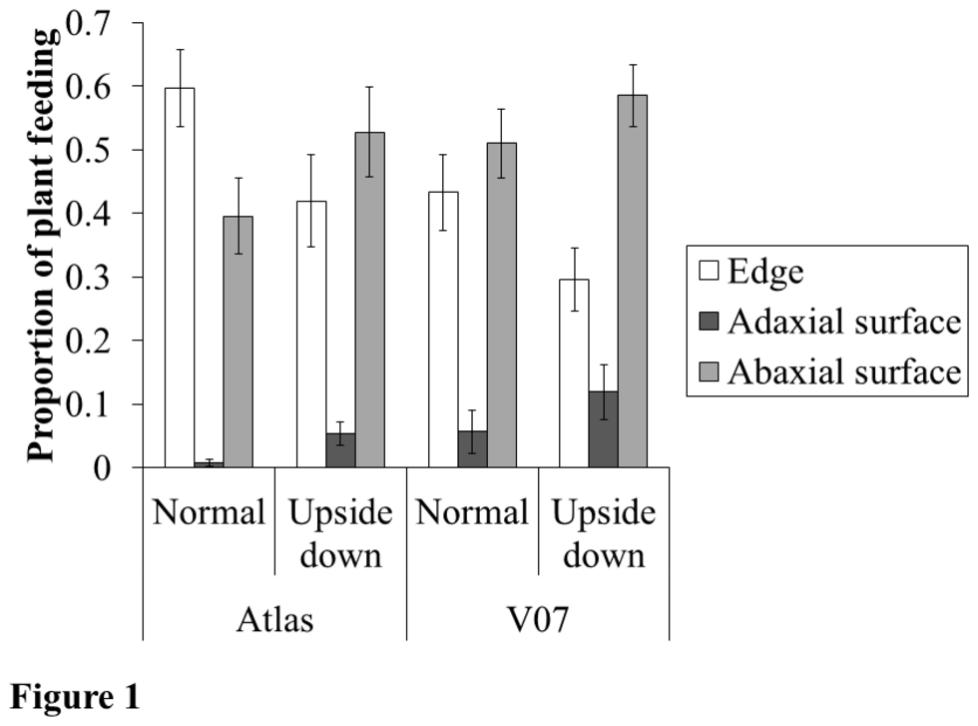
Feeding preferences of Western Flower Thrips on individual leaves. The feeding preferences of individual adult female Western flower thrips (WFT) were assessed on excised cotton cotyledons orientated ‘normally’ (i.e., abaxial-side down) and updside down (abaxial-side down) in Petri dishes. WFT were sealed for 3d with excised cotyledons from either one of two cotton genotypes (Atlas and V07).

On normally-oriented cotyledons (abaxial side-down), an aversion to adaxial-feeding by Western Flower Thrips (WFT) was highly significant on both genotypes based on Monte Carlo simulations (*P*<0.001; Figure 1), whereas the preference for feeding on the abaxial and edge varied depending on the plant genotype (Atlas: *P*<0.001, V07: *P*=0.138; Atlas: *P*=0.196, V07: *P*<0.001, respectively). By orienting the cotyledon upside down, the proportion of WFT feeding on the adaxial surface increased by ~4% (*F*_1, 110_=1.559, *P*=0.014), but the overall aversion to the adaxial surface persisted (*P*<0.001, each genotype) (Figure 1). Interestingly, when the cotyledon was up-side down WFT fed 11% more on the abaxial leaf surface (*F*_1, 110_=1.608, *P*=0.039) and 19% less on the edge (*F*_1, 110_=3.440, *P*=0.005) compared to a normally orientated cotyledon (Figure 1). 

### No-choice feeding and relative fitness bioassay

**Figure 2:**
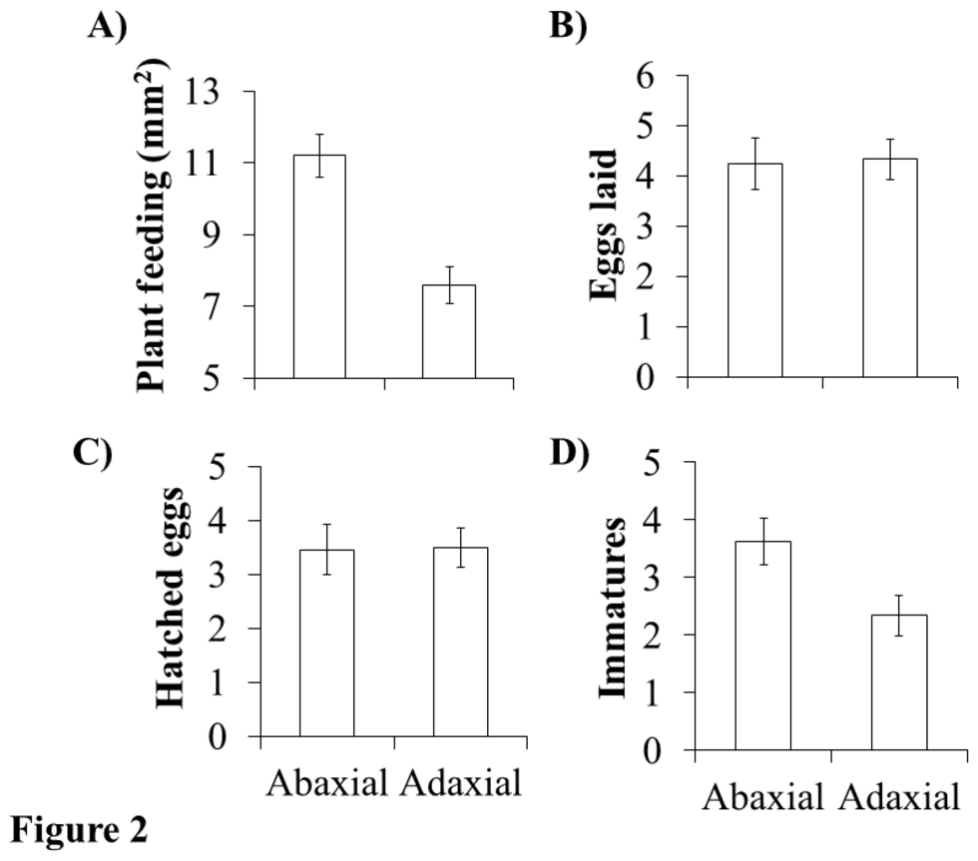
Effect of leaf surface on thrips feeding and reproduction. The effects of leaf surface on A) plant feeding (mm^2^), B) eggs laid, C) hatched eggs, and D) the number of alive immatures produced by an individual adult female Western Flower thrips during a 3d no-choice bioassay.

Under no-choice conditions WFT fed 32% less on the adaxial leaf surface compared to the abaxial surface (χ^2^=34.494, d.f.=1, *P*< 0.001; Figure 2A; Table 1). The reduction in feeding on the adaxial surface was due to fewer, large feeding scars: 33.5% fewer 2 mm^2^ feeding scars (χ^2^=6.720, d.f.=1, *P*= 0.009) and 59.1% fewer 3 mm^2^ feeding scars (χ^2^=11.330, d.f.=1, *P*< 0.001) compared to the abaxial surface. The final weight (χ^2^=0.001, d.f.=1, *P*= 0.423; Table 1), total number of eggs laid (χ^2^=0.024, d.f.=1, *P*= 0.917; Figure 2B; Table 1) and hatched eggs (χ^2^=0.004, d.f.=1, *P*= 0.963; Figure 2C; Table 1) did not vary between each leaf surface, but there were significantly fewer immatures recovered from the adaxial leaf surface (χ^2^=13.631, d.f.=1, *P*= 0.018; Figure 2D; Table 1). Plant feeding was a highly significantly covariate to the number of immatures emerging (χ^2^=59.736, d.f.=1, *P*< 0.001). Furthermore, the effect of leaf surface on immatures was highly significant using plant feeding as a covariate (χ^2^=13.631, d.f.=1, *P*= 0.009).

**Table 1:** GLM results for effects of cotton genotype, leaf surface (abaxial vs. adaxial), trial, initial weight on Western flower thrips A) plant feeding (mm^2^), B. eggs laid, C. hatched eggs, D. immatures recovered, and E. final weight (μm).

	**A) Plant feeding (mm^2^)**			**B) Eggs laid**			**C) Hatched eggs**			**D) Immatures recovered**			**E) Final weight**		
**Source of variation**	**df**	**Deviance**	**P**	**df**	**Deviance**	**P**	**df**	**Deviance**	**P**	**df**	**Deviance**	**P**	**df**	**Deviance**	**P**
Plant genotype (G)	1	0.549	0.531	1	3.650	0.204	1	4.216	0.155	1	0.032	0.909	1	<0.001	0.914
Leaf surface (S)	1	34.494	<0.001	1	0.024	0.917	1	0.004	0.963	1	13.631	0.018	1	<0.001	0.423
Trial (T)	1	20.526	<0.001	1	8.871	0.048	1	8.487	0.044	1	1.626	0.415	1	<0.001	0.256
Initial weight (W)	1	0.003	0.961	1	5.422	0.121	1	9.262	0.035	1	6.233	0.110	1	0.014	<0.001
G x S	1	0.443	0.574	1	4.681	0.150	1	4.472	0.131	1	3.185	0.254	1	<0.001	0.365
G x W	1	1.025	0.392	1	3.614	0.206	1	2.079	0.318	1	0.903	0.543	1	<0.001	0.947
G x T	1	5.418	0.049	1	6.148	0.099	1	4.263	0.153	1	0.084	0.853	1	<0.001	0.090
S x W	1	3.227	0.129	1	9.433	0.041	1	5.705	0.098	1	4.726	0.164	1	<0.001	0.147
S x T	1	0.160	0.736	1	3.206	0.234	1	3.630	0.187	1	0.337	0.711	1	<0.001	0.531
W x T	1	2.469	0.184	1	0.318	0.707	1	0.217	0.747	1	0.435	0.673	1	<0.001	0.100
G x S x T	1	0.176	0.723	1	3.368	0.222	1	6.021	0.089	1	2.916	0.275	1	0.002	0.028
G x S x W	1	0.955	0.409	1	<0.001	0.994	1	0.172	0.774	1	2.501	0.312	1	<0.001	0.094
G x T x W	1	0.590	0.516	1	0.002	0.972	1	0.031	0.903	1	0.673	0.600	1	<0.001	0.499
S x T x W	1	3.104	0.137	1	0.202	0.765	1	1.588	0.383	1	0.502	0.651	1	<0.001	0.751
G x S x T x W	1	0.037	0.871	1	0.376	0.683	1	0.627	0.583	1	0.238	0.755	1	<0.001	0.013

### Do leaf biomechanical properties inhibit thrips feeding?

**Figure 3:**
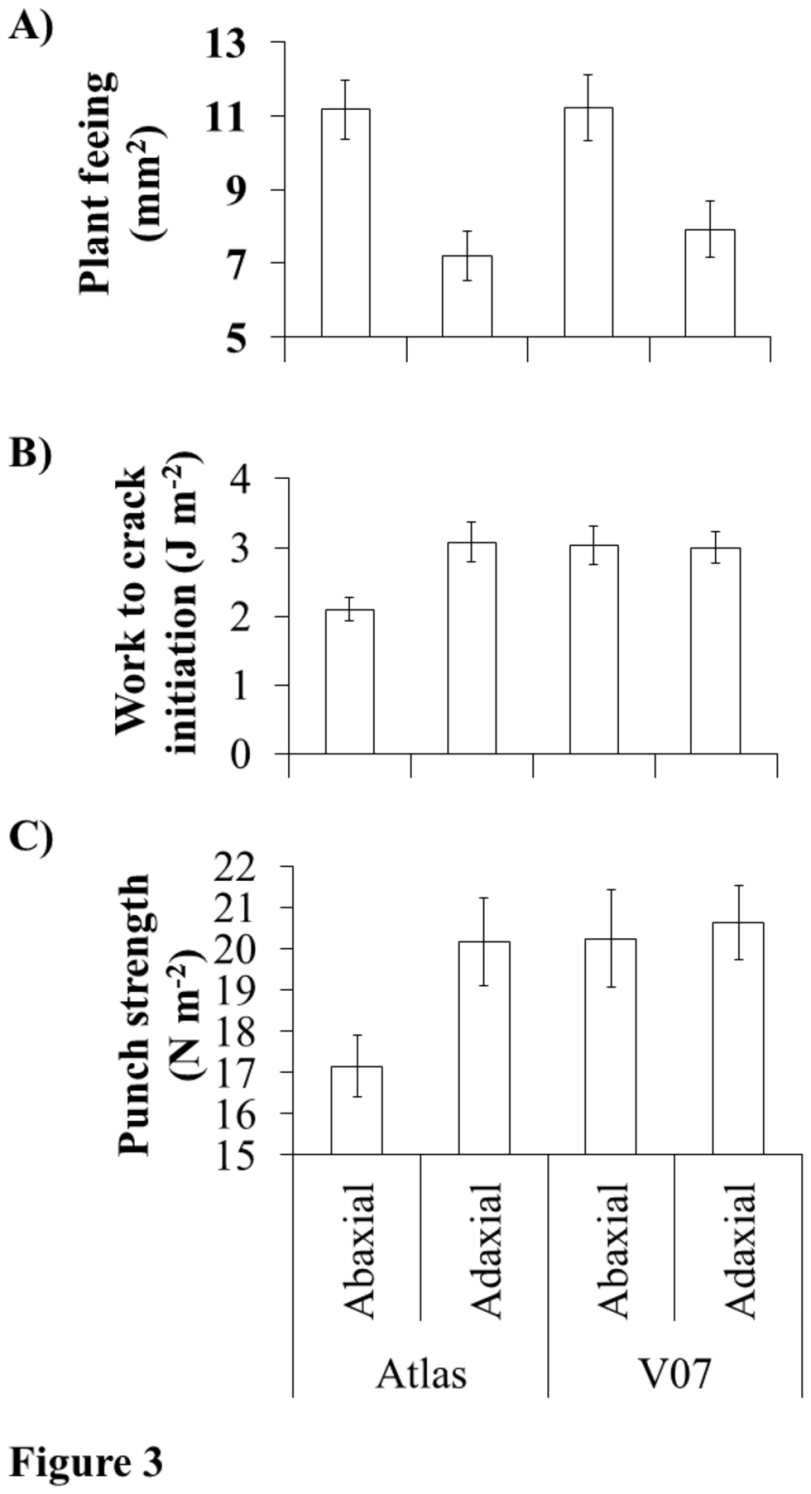
Effect of leaf surface and plant genotype on thrips feeding leaf biomechanical properties. Bar graphs illustrating the Effects of leaf surface (abaxial vs adaxial) and plant genotype (Atlas and V07) on thrips feeding (A) and two biomechanical properties of cotton cotyledons (*Gossypium hirsutum*): work to crack initiation (B) and punch strength (C). Mean plus SE is shown for each response variable.

Work to crack initiation was significantly affected by an interaction between plant genotype and leaf surface (χ^2^=5.167, d.f.=1, *P*=0.029) (Figure 3B). This was due to the abaxial surface requiring 31.7% less work to initiate a crack than the adaxial surface for the genotype Atlas, whereas there was no difference between leaf surfaces on genotype V05. While punch strength was not significantly affected by the interaction between genotype and leaf surface (χ^2^=1.783, d.f.=1, *P*=0.183), the general relationship was similar to that observed for work to crack initiation (Figure 3C). 

## Discussion

Our results showed that foraging of WFT on (normally-orientated) cotyledons was best characterized as an aversion to feeding on the adaxial surface rather than a preference for the abaxial surface. This is based on the evidence showing that the aversion to feeding on the adaxial surface was consistent among plant genotypes, whereas the preferences for abaxial and edge surfaces varied depending on plant genotype. Our first experiment investigated whether thrips aversion to feeding on the adaxial surface was related to an avoidance of direct exposure to light, i.e., negative-phototaxis. For this experiment the orientation of cotyledons was manipulated (normal vs up-side down), and if an aversion to light influenced thrips foraging then we predicted that on up-side down cotyledons thrips would feed predominantly on the adaxial surface. Two lines of evidence did not support this prediction. First, feeding on adaxial surface was characterized as a significant aversion relative to the edge an abaxial surfaces even when the cotyledon was up-side down. Second, manipulating the orientation of the cotyledon was associated with WFT feeding 11% more on the abaxial leaf surface (Figure 1). These results are difficult to reconcile under the assumption that negative phototaxis structures the foraging-decisions of WFT on individual cotton cotyledons, and suggest that other factors, possibly related to the properties of the cotyledon influenced WFT foraging. 

Another experiment investigated whether an aversion to adaxial-feeding could be attributed to the properties of the cotyledon itself. We predicted that if the aversion to adaxial-feeding was due to leaf properties then under no-choice conditions WFT would have lower performance on the adaxial surface than the abaxial surface. We found that WFT produced 35% fewer immatures on the adaxial surface (Figure 2D), which indicates clear effects on the relative fitness of adult WFT and establishes a preference-performance correlation that suggests the plant trait(s) causing the negative effects on thrips fitness (in no-choice conditions) also influence how thrips forage when given a choice. Furthermore, WFT feeding was 32% less on the adaxial surface compared to the abaxial (Figure 2A). Since the amount of feeding was positively associated with the number of immatures emerging it suggests that the reduction in resource consumption at least in part resulted in fewer immatures emerging from the adaxial surface. 

The use of plant feeding as a covariate did not change the significant effect of leaf surface on immatures, which suggests two additional possibilities, independent of resource consumption, for how leaf surface affected immatures emerging. First, the adaxial surface could affect immatures emerging through post-ingestive effects [11] on the mother thrips. Post-ingestive effects could be due to the nutritional or toxin profile of plant cells accessible from the adaxial surface, which affected the quality of progeny (active 1^st^ instars) of the female thrips (Figure 2D). The second possibility is that the adaxial surface was more difficult for 1^st^ instar thrips to emerge from regardless of the amount of resources consumed by the mother thrips. Thrips in suborder Terebrantia embed their eggs within the leaf tissue [13] and therefore the thickness of the upper epidermis or the cellular organization of the palisade layer might make it more difficult for 1^st^ instars to successfully emerge from within the leaf. 

**Figure 4:**
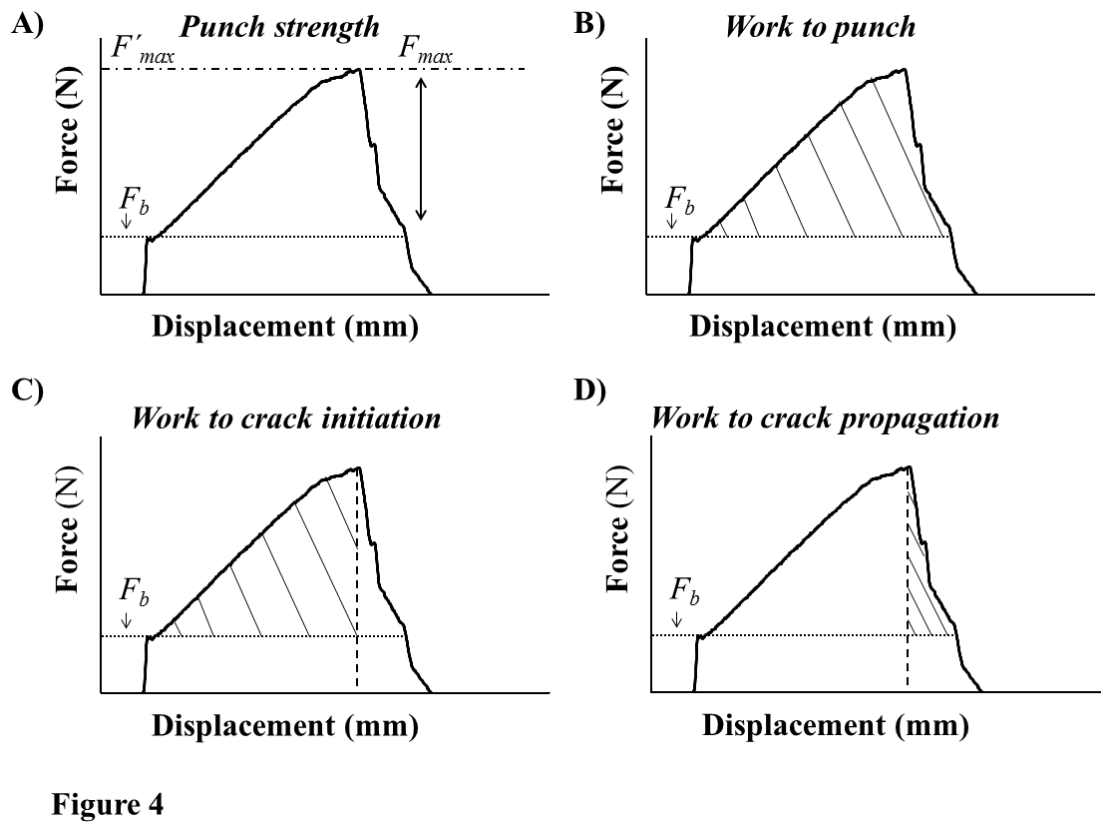
A visual representation of four leaf-biomechanical properties. Four biomechanical properties of cotton cotyledons (*Gossypium hirsutum* L.) were generated from a punch-and-die test (penetrometer). Most leaves were slightly curved and required force to initially flatten the leaf (base force, *F*_*b*_) on the die. When the punch started to compress the leaf, a sharp increase in force was observed. The leaf surface is assumed to crack at the maximum force (*F*_max_). A) ‘Punch strength’ is the maximum force (*F_max_*) (scaled to the area of the punch) required to initiate a crack in the leaf surface. B) ‘Work to punch’ is the total amount of work (i.e., area under curve) required to penetrate the entire leaf. We derived two additional properties called ‘Work to crack initiation’ (C) and ‘work to crack propagation’ (D) which represent the total amount of work required to initiate a crack in the leaf surface and the energy needed to propagate a crack through the leaf, respectively.

The evidence showing that WFT fed significantly less and produced fewer, large feeding scars (i.e. ≥2 mm^2^) on the adaxial leaf surface could indicate some sort of mechanical barrier inhibiting the size of thrips feeding scars. Therefore, our final experiment investigated the hypothesis that leaf biomechanical properties inhibited thrips feeding. Resistance to mandibular penetration could be particularly important for WFT because their mandible is fused proximally to the exoskeleton and requires generating an indirect force to penetrate the mandible through the leaf surface. For this experiment we examined the relationship between WFT feeding (mm^2^) and two leaf biomechanical properties that seemed particularly relevant to an intracellular feeder (Figure 4). We found that both biomechanical properties- one related to the peak force and the other to gross energy needed to fracture the leaf surface, showed a similar genotype X leaf surface effect. Importantly, these results provided only partial support for the biomechanical-hypothesis because on one cotton genotype (Atlas) there was a negative relationship between plant feeding and both biomechanical properties, but not on the other (Figure 3). These results in general are congruent with Peeters et al.. (2007) [14] who found that the leaf biomechanical properties of 18 co-occuring plant species were not correlated with the densities of shallow-suckers/chewers comprised of Thysanoptera, Diptera larvae, and grubs of unknown order. 

In this study, we systematically investigated why an intracellular feeding thrips showed an aversion to feeding on the adaxial surface of cotton cotyledons. We showed that feeding on the adaxial surface under no-choice conditions results in a reduction in relative fitness, which was at least in part due to fewer resources consumed on that surface. The reduction in feeding on the adaxial surface could be further attributed to fewer larger-sized feeding scars, suggesting that leaf properties that inhibit certain aspects of thrips feedings may underlie the aversion to adaxial feeding. Additional research that seeks to identify the leaf property that inhibits thrips feeding on the adaxial surface may not only unravel why thrips avoid foraging on the adaxial surface, but also could provide valuable insight for plant breeding programs that seek to enhance resistance in crop plants. 

## Materials and Methods

### Ethics Statement

This work did not involve endangered or protected species.

### General Procedures

Cotton plants were seeded individually in 125 ml pots (Metro-mix 900 [Sun Gro Horticulture, Bellevue, Washington, USA]) and cultivated in a small room (2.25m x 2.75m x 2.25m) (16:8 light:dark cycle, 13.1±5.2 μmol m^-2^ s^-1^, 34.7±11.1°C). For all experiments, cotton plants were 10-days old (from time of planting) at the time of experimentation. To minimize variation in plant quality between experiments, all experiments were conducted on the same day using plants randomly selected from a source 'batch'. This procedure was repeated during each of two trials. Adult female WFT were obtained from colonies maintained on bean cotyledons under florescent lights (12:12 light:dark, 1.2±0.3 μmol m^-2^ s^-1^, 25.0±3.3°C). The following two cotton genotypes were used in all experiments: 07-7-1001 hereafter referred to as “V07” which is an unreleased experimental breeding line developed by Texas AandM AgriLife Research Cotton Improvement Project, TX and All-Tex® Atlas (All-Tex Seed Inc., Levelland, TX; PVP: 9200188; PI 561579). These genotypes were selected based on preliminary experiments that indicated genotypic variation in the leaf biomechanical properties (see experiment 3 below). 

### Experiment 1: WFT feeding preferences (choice) and effects of leaf orientation

The first objective of this experiment was to document the foraging preferences of WFT on individual cotton cotyledons in terms of feeding on the edge, abaxial, and adaxial surfaces. The second objective was to evaluate whether the orientation of the cotyledon (i.e., abaxial side-down vs. abaxial side-up) altered such preferences. For this experiment, individual adult female WFT were sealed in 90-mm-diameter Petri dishes with an excised cotton cotyledon that was placed either abaxial side-down (‘normal’) or abaxial side-up (up-side down). Since the petiole ‘props-up’ the cotyledon when it is placed abaxial-side down in the petri-dish (‘normal’), toothpicks were placed under all cotyledons (in both treatment groups) to provide WFT access to the adaxial leaf surface when the cotyledon was up-side down. After three days, the total area of plant scarring by WFT on the edge, abaxial, and adaxial surface was quantified using a dissecting microscope and ocular micrometer and converted into proportions prior to analysis. Thrips produce feeding scars characterized as round silvery depressions that occur on the leaf surface and along the leaf edges. On the leaf surface specifically, WFT produce feeding scars that range from 1- to 3-mm^2^ in size, and the number of each sized feeding scar produced was used to extrapolate the total area of feeding on the abaxial and adaxial surface. If WFT females were missing, died, or did not feed during the course of the experiment the replicate was omitted from the analysis. 

The experimental design of the bioassay was a full factorial design with cotton genotype (V07 and Atlas) and leaf orientation (abaxial side-up vs adaxial side-down) as treatments (*n* =10-14 replicates for each treatment combination, each trail). For the first objective of this experiment, we used Monte Carlo resampling methods (10,000 iterations) to establish whether WFT showed a significant feeding preference or aversion to feeding on the edge, abaxial, and adaxial surfaces. Permutations were performed using the PopTools 3.2.5 extension for MS-Excel [15], and probability values were obtained based on the number of times re-shuffling the data resulted in means significantly larger (i.e., preference) or smaller (i.e., aversion) than the actual means. The remaining statistical analyses, in this experiment and in the two experiments described below, were performed with the statistical software package R [16]. The proportion of feeding on the abaxial, adaxial, and edge were each non-normally distributed (*P* <0.05, Shapiro-Wilks test). Therefore a Generalized Linear Model was used, which extends the range of application of linear statistical models to include response variables with non-normal distributions [16]. Specifically, the GLM was used tto investigate the effect of leaf orientation onthe proportion of feeding on the abaxial, adaxial, and edge. The model had a quasi-binomial error structure (due to over dispersion) and was analyzed using an F test [17]. 

### Experiment 2: No-choice feeding and relative fitness bioassay

To assess whether the quantity and/or quality of resources varied between each leaf surface, a no-choice leaf-disc bioassay was developed that restricted WFT to feeding only on either the abaxial or adaxial leaf surface. Leaf discs were generated using an open-ended copper pipe (diameter 2.5cm), and were placed individually in polyethylene-caps (diameter 2.5cm) of a vial (diameter 2.29cm, height 5.4cm) either abaxial side-up or abaxial side-down. Upon connecting the vial to the cap, a seal was created around the leaf disc by the edge of the vial that restricted the access of WFT to only one side of the leaf disc. 

The protocol for this experiment was as follows. An individual adult female was first weighed (μg) (Mettler-Toledo Ch-8606, Laboratory and Weighing Technologies, Greifensec, Switzerland) and then sealed in the vial (with leaf disc) for 3d. After three days, individual thrips were removed, re-weighed and the number of 1-, 2-, and 3mm^2^ feeding scars on the leaf disc was tallied, and the total area of feeding scars (mm^2^) was extrapolated from these values. Next, leaf discs were re-sealed in the vial without the adult female thrips for an additional three days to allow viable thrips larvae to hatch from eggs laid within the leaf disc (thrips in suborder Terebrantia lay eggs in leaf tissue [13]). After three days, the number of live immature thrips (hereafter referred to as ‘immatures’) on the leaf disc was quantified. Last, to quantify the number of eggs laid and hatched eggs, the leaf disc was stained with two solutions as per [18]. The first solution consisted of 0.2% acid fuchsin (the staining agent) mixed in equal portions of 95% ethanol and glacial acetic acid. The second solution enhanced the leaf disc’s transparency and consisted of equal portions of distilled water, 99% glycerine, and 85% lactic acid. The staining process allowed thrips eggs to be seen under a dissecting microscope, which were identified as kidney-shaped with hatched-eggs having a red transparent appearance and unhatched eggs appearing dark-red. If the adult female thrips were missing, died, or did not feed during the course of the experiment, the replicate was omitted from the analysis. 

The experimental design of the leaf-disc bioassay was a full factorial design with plant genotype (V07 and Atlas) and leaf surface (under-surface vs upper-surface) as treatments (*n* =11-14 replicates for each treatment combination and each trial). The area and size of feeding scars, and various performance measures (eggs laid, hatched eggs, immatures recovered, and final weight) were non-normally distributed (*P* <0.05, Shapiro-Wilks test). Therefore, the effect of trial, leaf surface and plant genotype were analyzed using a Generalized Linear Model (Quasi-poisson error structure due to over dispersion) with a Chi square test with initial weight, and in some cases plant feeding as covariates [11,17]. The use of plant feeding as a covariate was used to tease apart whether leaf surface effects on performace were due to pre- vs. post-ingestive effects [11]. 

### Experiment 3: Do leaf biomechanical properties influence WFT feeding?

No-choice results indicated that feeding was significantly reduced on the adaxial surface (see results section), which lead us to investigate whether leaf biomechanical properties inhibit WFT feeding. Therefore, the goal of this experiment was to investigate the relationship between leaf biomechanical properties and WFT feeding. To investigate leaf biomechanical properties, a penetrometer was used to measure the force required to pass a blunt punch (or rod) through the abaxial and adaxial surface of a cotyledon. Given the lack of published data on how much force or energy is required for thrips to gain access to the intracellular contents of plants, we assume that the leaf biomechanical properties measured using a penetrometer are relevant indicators of the mechanical properties that thrips contend with during a feeding bout. Additionally, the biomechanical properties derived from penetrometers have been previously investigated as potential feeding deterrents for various herbivore feeding guilds, including intracellular feeders, which were referred to as ‘sallow suckers’ in [14]. 

The punch, a flat-ended, steel cylinder (diameter=1.5mm), and die (diameter 3.175mm) were installed into a general testing penetrometer (Model TAXT2i, Texture Technologies Corp., Scarsdale, NY, USA). The punch speed was kept constant (0.2 mm s^-1^) and the machine simultaneously recorded load (N) applied to the sample and displacement (mm) of the punch (every 0.005 s). Because the penetrometer is destructive, we sampled the biomechanical properties from representative cotyledons and correlated these results with WFT feeding data derived from no-choice conditions. The penetrometer data were collected on the same day that the WFT bioassay was initiated using the same batch of plants. Effort was made to avoid major veins and to sample the biomechanical properties from roughly the same location on the cotyledon as to where the center of each leaf disc would have been [12]. 

A penetrometer generates force-displacement curves, which were used to derive two biomechanical properties: punch strength and work to crack initiation (Figure 4, Table 2). Punch strength is the maximum force needed to initiate a crack in the leaf surface [12] (Figure 4A, and Table 2), which could be a relevant measure with respect to the feeding of sap-sucking herbivores, as well as for chewers, because each must first initiate a crack in the leaf surface in order to access and consume plant materials beneath. Because work to punch (Figure 4B) would seem to overestimate the energy required during a feeding bout for sap-sucking herbivores (but not for chewers), a new property was derived that represents the work needed to initiate a fracture in the leaf surface, aptly named ‘work to crack initiation’ (Figure 4C, and Table 2). Our derivation assumes that work to punch can be viewed as a composite measure of two events, work to crack initiation and work needed to propagate the crack through the leaf (Figure 4C,D and Table 2). 

**Table 2:** Leaf biomechanical properties, their derivation, and the herbivore feeding guild potentially affected.

Leaf biomechanical property	Calculation	Herbivore feeding guild potentially affected
Punch strength (Figure 1A)	(*F*_*max*_ -*F*_*b*_)/*A*	Piercing-sucking and chewing
Work to punch (Figure 1B)	∫ [(*F*_*x*_-*F*_*b*_)*D*_*x*_]/*A*	Chewing
Work to crack initiation (Figure 1C)	∫ [(*F*_*y*_-*F*_*b*_)*D*_*y*_]/*A*	Piercing-sucking and chewing

*F*_*max*_, maximum force (N); *F*_*b*_, force needed to flatten the cotyledon against the die; A, area of punch (m^2^); *F*_*x*_, force and *D*_*x*_, displacement (mm) at any point, *x*, between initiation of leaf compression and complete fracture of cotyledon; *F*_*y*_, force and *D*_*y*_, displacement at any point, y, between initiation of leaf compression and the maximum force (*F*_*max*_).

The leaf biomechanical assay was a full factorial design with plant genotype (V07 and Atlas) and leaf surface (under-surface vs upper-surface) as treatments (*n* =23-30 replicates for each treatment combination, each trial). In general, normality could not be achieved and therefore a generalized linear model error was generated with a quasipoisson error structure due to over dispersion. The model was analyzed with a Chi square test to determine the effects of plant genotype, leaf surface, and trial on punch strength and work to crack initiation [17]. 
